# Near-infrared spectroscopy as a predictor of clinical deterioration: a case report of two infants with duct-dependent congenital heart disease

**DOI:** 10.1186/s12887-017-0839-3

**Published:** 2017-03-16

**Authors:** Mirthe J. Mebius, Gideon J. du Marchie Sarvaas, Diana W. Wolthuis, Beatrijs Bartelds, Martin C. J. Kneyber, Arend F. Bos, Elisabeth M. W. Kooi

**Affiliations:** 1 University Medical Center Groningen, Beatrix Children’s Hospital, Division of Neonatology, University of Groningen, Hanzeplein 1, 9713 GZ Groningen, The Netherlands; 2 University Medical Center Groningen, Beatrix Children’s Hospital, Division of Pediatric Intensive Care, University of Groningen, Groningen, The Netherlands; 3 University Medical Center Groningen, Center for Congenital Heart Diseases, Pediatric Cardiology, Beatrix Children’s Hospital, University of Groningen, Groningen, The Netherlands; 4 Critical Care, Anesthesiology, Peri-operative & Emergency medicine (CAPE), the University of Groningen, Groningen, The Netherlands

**Keywords:** Congenital heart disease, Near-infrared spectroscopy, Newborn infants, Cardiac arrest, Cardiopulmonary resuscitation, Intensive care, Case report

## Abstract

**Background:**

Some infants with congenital heart disease are at risk of in-hospital cardiac arrest. To better foresee cardiac arrest in infants with congenital heart disease, it might be useful to continuously assess end-organ perfusion. Near-infrared spectroscopy is a non-invasive method to continuously assess multisite regional tissue oxygen saturation.

**Case presentation:**

We report on two infants with duct-dependent congenital heart disease who demonstrated a gradual change in cerebral and/or renal tissue oxygen saturation before cardiopulmonary resuscitation was required. In both cases, other clinical parameters such as heart rate, arterial oxygen saturation and blood pressure did not indicate that deterioration was imminent.

**Conclusions:**

These two cases demonstrate that near-infrared spectroscopy might contribute to detecting a deteriorating clinical condition and might therefore be helpful in averting cardiopulmonary collapse and need for resuscitation in infants with congenital heart disease.

## Background

Some infants with congenital heart disease (CHD) are at risk of cardiac arrest during hospitalization [1]. In-hospital cardiac arrest occurs in approximately 2% to 6% of children admitted to paediatric intensive care units [2, 3]. Through earlier recognition, cardiac arrest may be prevented and outcomes and survival may be improved [4].

Monitoring end-organ perfusion might help recognizing clinical deterioration at an early stage. Near-infrared spectroscopy (NIRS) is a non-invasive clinical tool to continuously assess multisite regional tissue oxygen saturation (rSO_2_) [5]. It is based on the relative transparency of biological young tissue (bone, skin, soft tissue) and the ability to differentiate oxygenated hemoglobin from deoxygenated hemoglobin as they have distinct absorption spectra. The ratio between oxygenated and total hemoglobin represents the regional tissue oxygen saturation [6]. In infants with CHD, multiple studies have investigated tissue oxygenation at various moments during early life [7–14]. Most studies addressed the intraoperative and postoperative period [7–10], but cerebral rSO_2_ was also assessed preoperatively in some studies [11–14]. To our knowledge, however, multisite tissue oxygen saturation as a predictor of clinical deterioration in neonates with CHD has not been described before.

We discuss two cases in which continuous monitoring of multisite tissue oxygen saturation using NIRS indicated a deteriorating clinical condition which eventually led to cardiac arrest.

## Case presentation 

Both cases in this report were part of a prospective observational cohort study (registration number NTR5233), which was conducted between May 2014 and August 2016. Parental informed consent was obtained in both cases. We measured cerebral and renal rSO_2_ using INVOS 5100c near-infrared spectrometers (Somanetics Corporation, Troy, Michigan, USA) in combination with neonatal sensors (Somanetics Corporation). Nurses were not instructed to act on certain rSO_2_ values, as they were not measured for clinical purposes.

### Case 1

A boy diagnosed prenatally with pulmonary atresia with an intact ventricular septum was born at our hospital at a gestational age of 37.3 weeks. After birth, the patient was admitted to the neonatal intensive care unit (NICU) with continuous positive airway pressure (CPAP) at seven cmH_2_O with a maximum of 40% O_2_ and infusion with prostaglandin E_1_ (0.025 ug/kg/min) to maintain pulmonary circulation. Prenatal diagnosis and ductal patency were confirmed by the pediatric cardiologist. The patient appeared to be hemodynamically stable with transcutaneous oxygen saturations (SpO_2_) between 70% and 90% and mean arterial blood pressures between 46 and 73 mmHg. As the patient was clinically stable, further hemodynamic parameters were not available for this case.

Four hours after birth the boy developed three apneas. The third apnea was followed by loss of cardiac output. Cardiopulmonary resuscitation was initiated immediately according to protocol. Cardiac output and heart rate, however, remained insufficient and the boy died 6 h after birth.

Autopsy confirmed the diagnosis of a pulmonary atresia with a hypoplastic right ventricle and an intact ventricular septum. Furthermore, autopsy revealed a single coronary ostium, multiple ventricular-coronary fistulas and a right ventricle dependent coronary blood flow with bridging of a branch of the right coronary artery. There were signs of recent myocardial ischemia, such as loss of cardiomyocytes. In addition, there was a left-sided open ductus arteriosus between the brachiocephalic trunk and the pulmonary artery. Based on autopsy findings, the most likely cause of death was considered to be massive myocardial ischemia which is not uncommon for this type of anatomy and physiology. Other causes such as a primary intraventricular hemorrhage or infarction cannot be ruled out completely since there was no permission for cerebral autopsy.

### Near-infrared spectroscopy case 1

**Fig. 1:**
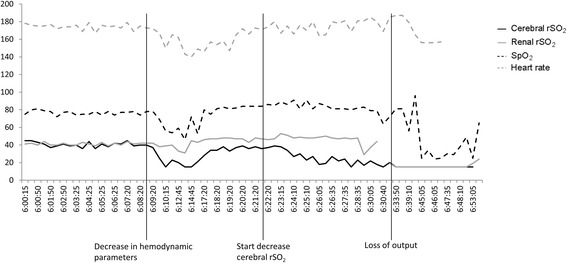
The course of hemodynamic parameters and cerebral and renal oxygen saturation of the first case. SpO_2_, transcutaneous arterial oxygen saturation; rSO_2_, regional tissue oxygen saturation

Baseline cerebral and renal rSO_2_ were relatively low (around 40%) and decreased rapidly approximately 40 min before cardiopulmonary resuscitation was necessary (Fig. 1). This rapid decrease in cerebral and renal rSO_2_was probably due to a sudden decrease in arterial oxygen saturation. All clinical parameters spontaneously recovered from this incident. Cerebral rSO_2_, however, started decreasing again from approximately 40% to 15% 25 min before cardiopulmonary resuscitation was necessary, while other hemodynamic parameters did not alarm the attending staff. Within 10 min, cerebral rSO_2_ had already decreased 50% from its original baseline value (40%) to approximately 20%. We cannot fully explain why cerebral rSO_2_ decreased before renal rSO_2_ and SpO_2_.

### Case 2

A girl diagnosed prenatally with double outlet right ventricle, transposed great arteries, a ventricular septal defect and a hypoplastic aortic arch was born prematurely at our hospital at a gestational age of 34.9 weeks. She was admitted to the NICU with CPAP 10cmH_2_O with a maximum of 40% O_2_ due to respiratory distress/delayed transition. Initially, she had a wide-open ductus arteriosus. Eighteen hours after birth her clinical condition deteriorated with signs of a duct-dependent systemic circulation. Therefore, prostaglandin E_1_ infusion was administered (0.025 ug/kg/min) to maintain ductal patency. During the first days after birth there was a fragile balance between pulmonary and systemic blood flow.

At the sixth day after birth, she underwent bilateral pulmonary banding with the intention of controlling an ever increasing pulmonary blood flow. Peri-operatively, hypotension was treated successfully with multiple volume expansions and noradrenalin. Postoperatively, she was on Bi-level Positive Airway Pressure (BiPAP)-assist and prior to loss of output there were no clinical signs of primary respiratory failure (e.g. inadvertent extubation). Arterial pH was 7.34, arterial saturation was 90%, pCO_2_ was 4.8 kPa, hemoglobin was 8.0 mmol/l and lactate was 1.5 mmol/l. At that time, arterial blood pressure was 70/30 mmHg and she had normal diuresis. One hour later, she suddenly developed bradycardia followed by loss of cardiac output. Cardiopulmonary resuscitation was initiated immediately according to protocol. Infusion with prostaglandin E_1_ was transferred from a peripheral venous site to a central line and was increased in dosage. An echocardiogram suggested an open ductus arteriosus, revealed a poor contractility and an inadequate cardiac output. After 40 min of cardiopulmonary resuscitation, the sternum was re-opened. The heart, however, was almost non-contractile and the girl died 7 days after birth.

Autopsy confirmed the diagnosis and concluded that the lumen of the ductus arteriosus was small with a diameter of less than three millimeter. Whether this was too small to maintain systemic circulation and whether prostaglandin E_1_ infusion was inadequate at the time of collapse, remains unknown. Furthermore, autopsy revealed an immature lung parenchyma, atelectasis of part of the basal lobes and small inflammatory areas in both lungs. These pulmonary findings might also have contributed to the cardiopulmonary collapse.

### Near-infrared spectroscopy case 2

**Fig. 2:**
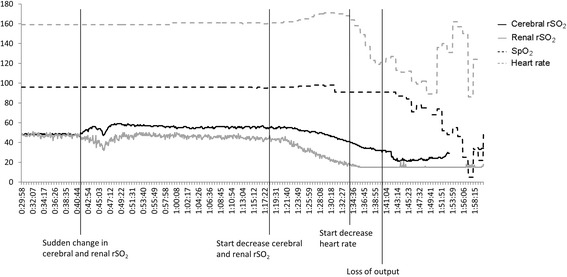
The course of hemodynamic parameters and cerebral and renal oxygen saturation of the second case. SpO_2_, transcutaneous arterial oxygen saturation; rSO_2_, regional tissue oxygen saturation

We observed an increase in cerebral rSO_2_ and a decrease in renal rSO_2_ 1 h before loss of output (Fig. 2). Renal rSO_2_ returned to baseline, while cerebral rSO_2_ remained approximately 10% above baseline. This incident might have been caused by a closing ductus arteriosus due to insufficient Prostaglandin E_1_ administration or an insufficient response to adequate Prostaglandin E_1_ administration. Especially the rapidly decreasing renal rSO_2_ was indicative of a closing ductus arteriosus. Due to the coarctation of the aorta, renal blood flow was dependent on an open ductus arteriosus. The increase in cerebral rSO_2_ was less easy to explain, but could be caused by an increase in cerebral blood flow. Due to the closing ductus arteriosus, the descending aorta might have become narrower, leading to an increased arterial pressure above the coarctation and subsequently increased cerebral blood flow. Thirty minutes before cardiopulmonary resuscitation, cerebral rSO_2_ started decreasing from 55% to approximately 20% and renal rSO_2_ decreased from 40% to 15%. Renal rSO_2_ decreased more rapidly compared with cerebral rSO_2_ and approximately 15 min before loss of output, renal rSO_2_ was less than 50% from its original baseline value. The more rapid decrease of renal rSO_2_ in comparison with cerebral rSO_2_ also supports the theory of insufficient systemic circulation due to a closing ductus arteriosus. Apart from a slowly increasing heart rate from 160 bpm to 180 bpm, hemodynamic parameters such as SpO_2_ and arterial blood pressure remained stable until several minutes before cardiopulmonary resuscitation was necessary (Fig. 2).

## Discussion

These cases demonstrate that continuous monitoring of multisite near-infrared spectroscopy might be helpful in early identification of clinical deterioration in infants with congenital heart disease. In both cases, cerebral and/or renal rSO_2_ changed 30 min or more before loss of output while other hemodynamic parameters did not indicate that deterioration was imminent until a few minutes before cardiopulmonary resuscitation was necessary.

Cerebral rSO_2_ decreased 30–35% from baseline before loss of output. McNeill et al. found that cerebral rSO_2_ values varied 15% or more from baseline rSO_2_ values during less than 1% of the day in preterm infants [15]. Furthermore, Bernal et al. demonstrated an average cerebral rSO_2_ of 77% with a standard deviation of 6.1% within patients during the first week after birth [16]. As a decrease of 30–35% exceeds this intra-individual variability, it seems more likely that the decrease in cerebral rSO_2_ observed in the two cases was a consequence of a deteriorating clinical condition and a subsequent reduction of cerebral perfusion, rather than the normal intra-individual variability of NIRS.

In the current cases we did not respond to changes in cerebral and renal rSO_2_, as they were measured in the context of a prospective observational cohort study and not for clinical purposes. In retrospect, these cases indicate that large changes in cerebral and/or renal rSO_2_ in infants with duct-dependent CHD should be considered a red flag and warrant a complete check-up of the infant, including physical examination, echocardiography and basic blood tests. The time-interval of 15 to 30 min might give some extra time to prevent clinical deterioration in infants with CHD. Potential therapeutic strategies depend on the reason of the change in cerebral and/or renal rSO_2_, but could include mechanical ventilation, pharmaceutical therapy (e.g. inotropes), volume expansion or perhaps emergency surgery. Furthermore, it might be useful to use predefined upper and lower limits of cerebral and renal oxygen saturation, a principle that was recently suggested in a large trial with preterm infants [17]. Due to the heterogeneity of the cardiac population, it is difficult to apply normal ranges of cerebral and renal rSO_2_. In this population it might be more useful to use limits below which histological and biochemical changes in tissue have been reported, i.e. below 44% [18].

## Conclusions

In conclusion, in infants with duct-dependent congenital heart disease, a significant change in cerebral and/or renal oxygen saturation from baseline as measured by NIRS may be an early indicator of a sudden, unexpected clinical deterioration, which eventually could lead to cardiac arrest and death. Increased awareness and thorough scrutiny of the patient are required when such changes in cerebral and/or renal oxygen saturation are observed.

## Abbreviations

BiPAP-assist: Bi-level Positive Airway Pressure - assist
CHD: Congenital heart disease
CPAP: Continuous positive airway pressure
NICU: Neonatal intensive care unit
NIRS: Near-infrared spectroscopy
rSO_2_: Regional tissue oxygen saturation
SpO_2_: Transcutaneous arterial oxygen saturation
